# Applications and Research Progress of Aerogels in Fire-Resistant Coatings

**DOI:** 10.3390/polym17202777

**Published:** 2025-10-17

**Authors:** Haitao Yang, Shouyan Guo, Kejia Kang, Mengjie Zhao, Fan Zhang, Xuexun Guo, Weigao Qiao, Gangfeng Tan

**Affiliations:** 1 Hubei Longzhong Laboratory, Xiangyang 441106, China; yanghaitao@lz-lab.com (H.Y.); zhaomengjie@lz-lab.com (M.Z.); zhfan@whut.edu.cn (F.Z.); 2 College of Mechanical and Electrical Engineering, Henan University of Technology, Zhengzhou 450001, China; 2024930873@stu.haut.edu.cn; 3 State Key Laboratory of Materials Composite Technology, Wuhan University of Technology, Wuhan 430070, China; 4 Industrial Research Institute, Suizhou Wuhan University of Technology, Suizhou 441300, China; guo6531@163.com (X.G.); qwg@126.com (W.Q.); auto8vum@whut.edu.cn (G.T.)

**Keywords:** aerogel, fire retardant coating, thermal insulation, surface modification, fire performance, structure design

## Abstract

This review establishes a comprehensive technical framework for aerogel-based fire-resistant coatings, from fundamental mechanisms to industrial applications. It analyses the multi-mode flame-retardant and thermal insulation mechanisms achieved through aerogels’ synergistic suppression of heat conduction, convection, and radiation, establishing their theoretical basis. The work compares the intrinsic characteristics of silica-based, carbon-based, and bio-based aerogels, providing rational selection criteria for fire protection systems. The study examines key integration challenges: balancing nanopore preservation with interfacial compatibility, inherent mechanical weaknesses, conflicts between high filler loading and workability, and scalability issues. It evaluates targeted strategies including interface engineering, mechanical reinforcement, workability optimization, and low-cost production routes. Application prospects in construction, tunneling, and cable protection are outlined. This review provides a coherent progression from mechanisms and material properties to challenges and solutions, offering theoretical guidance and a technical roadmap for developing next-generation high-performance fire-resistant coatings.

## 1. Introduction

**Figure 1:**
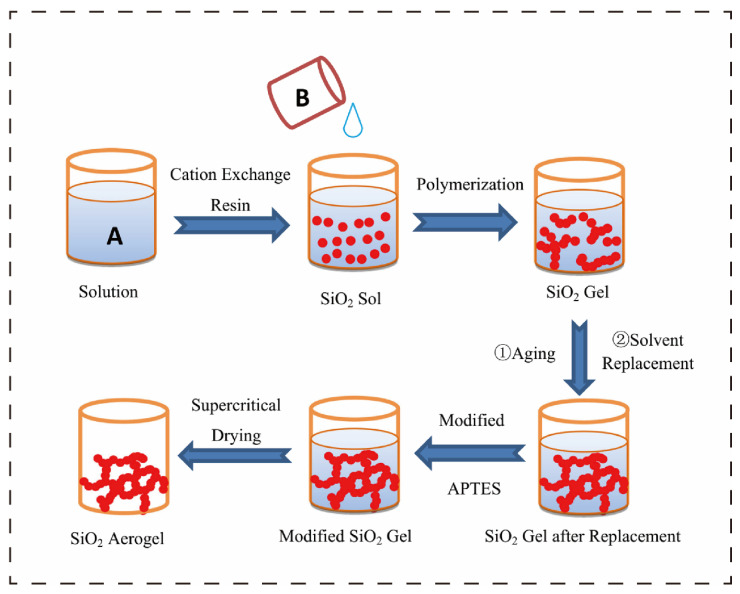
Schematic diagram of the SiO_2_ aerogel [12]. A is water glass solution, B is NaOH solution.

Aerogels are a class of porous solid materials composed of nanoscale frameworks. Their unique three-dimensional network structure endows them with high porosity (typically >90%), ultra-low bulk density (as low as 10 mg cm^−3^), and excellent thermal insulation performance (thermal conductivity usually <20 mW·m^−1^·K^−1^), making them one of the lightest solid materials known to date [1,2]. Based on raw material sources and chemical compositions, they can be categorized into inorganic aerogels (e.g., silica-based, carbon-based, and metal oxide-based) [3,4,5], organic aerogels (e.g., polyurethane-based and polyimide-based) [6], and bio-based aerogels (e.g., sodium alginate-based, cellulose-based, and chitosan-based) [7,8]. Among these, SiO_2_ aerogels are the most extensively researched, widely applied type to date [9]. The preparation process mainly involves three steps: first, forming a wet gel through a sol–gel reaction (solute molecules form a three-dimensional network via hydrolysis and condensation, encapsulating the solvent); then, removing the solvent using freeze-drying or supercritical drying techniques (to avoid skeleton collapse caused by conventional drying); and for some systems, further optimizing the microstructure and macroscopic properties through chemical cross-linking (e.g., silanization) or physical regulation (e.g., directional freeze-casting). Figure 1 shows a schematic diagram of the SiO_2_ aerogel. Benefiting from these characteristics, aerogels exhibit tremendous application potential in fields such as low-temperature thermal management (cold chain transportation and thermal insulation for polar equipment) [8], high-temperature fire protection (industrial pipeline protection and building wall flame retardancy) [10], environmental governance (organic pollutant adsorption) [7], new energy safety (battery thermal runaway suppression) [4], and aerospace (thermal protection components for spacecraft) [11].

Among diverse application domains, the fire-resistant coatings sector exhibits particularly pronounced demands for aerogels, serving as a significant driver for aerogel material R&D and application expansion. With increasingly stringent fire safety regulations, industries including construction, transportation, and industrial manufacturing impose heightened requirements for thermal insulation performance, flame-retardant efficiency, and durability in fire-resistant coatings. Aerogels—leveraging their exceptional thermal insulation and lightweight properties—precisely align with the core requirements of blocking heat transfer and delaying substrate combustion under high-temperature conditions. This positions aerogels as pivotal candidate materials for enhancing coating performance. Crucially, practical demands within this sector substantially guide development trajectories in aerogel performance optimization, cost containment, and application compatibility.

Nevertheless, aerogels face significant challenges in fire-resistant coating applications and cannot function as stand-alone materials. This limitation stems primarily from mismatches between their inherent properties and practical requirements: First, while pristine aerogels exhibit exceptional thermal insulation and lightweight characteristics, their poor mechanical properties—high brittleness and inadequate fatigue resistance—render them vulnerable to structural failure under mechanical stresses (e.g., vibration and compression) in complex environments. Second, functional singularity restricts the application scope; for instance, in fire protection, pure aerogels primarily rely on physical barrier effects and lack chemical flame-retardant components, thus proving insufficient for high-efficiency fire suppression during prolonged high-temperature combustion or complex fire scenarios. Additionally, relatively high manufacturing costs and insufficient interfacial bonding with other materials make individual applications prone to failure. Consequently, aerogels typically require integration with polymer matrices and flame retardants, leveraging synergistic effects to mitigate deficiencies and expand applicability.

Whilst the application of aerogels in fire-resistant coatings has garnered significant attention, the existing research often remains fragmented, focusing on isolated aspects such as material synthesis or performance optimization. To address this, the present review endeavors to establish a systematic analytical framework that interlinks the fundamental mechanisms, material selection criteria, technical challenges, composite strategies, and practical applications of aerogel-based fire-retardant coatings into a coherent whole. This work begins by elucidating the multi-faceted flame-retardant mechanisms, followed by a systematic comparison of the intrinsic properties of various aerogel types—including silica-based, carbon-based, and bio-based variants—to establish rational selection criteria for fire-retardant systems. It further provides a critical examination of the core challenges encountered during coating formulation, such as interfacial compatibility, mechanical reinforcement, application techniques, and scalable production costs, alongside an evaluation of advanced composite modification strategies. Finally, the review outlines future prospects and emerging trends in key sectors including construction, tunneling, and high-end equipment manufacturing. By following this “mechanism–material–challenge–strategy–application” trajectory, this review aims to clarify the intrinsic structure–property–application relationships, thereby offering a systematic reference for identifying critical scientific issues, optimizing material design approaches, and facilitating the targeted development of high-performance products.

## 2. Function Mechanisms of Aerogels in Fire-Resistant Coatings

### 2.1. Multiple Mechanisms of Aerogels Themselves

The core functionality of aerogels in fire-resistant coatings is rooted in their unique nanoporous solid structure, which enables the construction of an exceptionally efficient barrier against energy and mass transfer. Their mechanism is far more complex than that of traditional materials, manifesting primarily through synergistic thermal insulation and physical flame retardancy.

**Figure 2:**
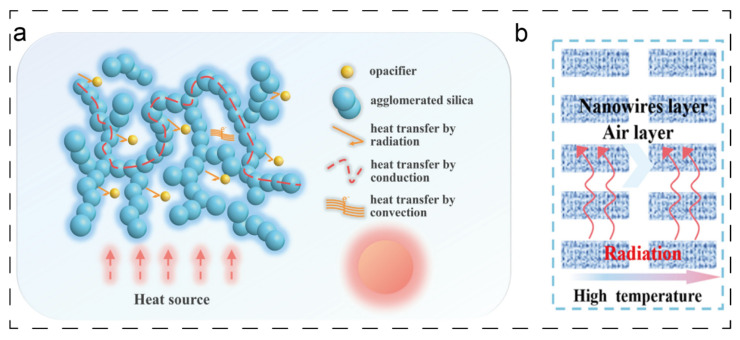
TiO_2_-enhanced radiation shielding [16]: (**a**) thermal insulation mechanism of SA/TiO_2_ aerogels; (**b**) schematic illustration of heat transfer pathways.

Their exceptional thermal insulation performance stems fundamentally from the cooperative suppression of the three primary heat transfer pathways: conduction, convection, and radiation. Firstly, the nanoscale pores (typically <70 nm) within aerogels induce a pronounced Knudsen effect [9,13,14]: as the pore diameter is smaller than the mean free path of air molecules, the gas molecules become confined, virtually eliminating convective heat transfer. This phenomenon is the key differentiator between aerogels and all conventional thermal insulation materials. Secondly, concerning the inhibition of heat conduction, the inherently very low volume fraction of the solid skeleton drastically restricts the available pathways for heat flow. Furthermore, as heat travels through the delicate nano-network skeleton, its path is considerably prolonged. Crucially, the nanoscale diameter of the skeleton induces significant phonon scattering [15], thereby reducing solid-phase thermal conductivity to an extremely low level. Furthermore, to counter radiative heat transfer, which becomes dominant at high temperatures, infrared opacifiers (such as TiO_2_) can be incorporated directly into the aerogel skeleton during its synthesis. This integration endows the aerogel with an inherent ability to effectively reflect and scatter infrared radiation, thereby preserving its insulation superiority across the entire temperature range [16,17]. A schematic illustrating this TiO_2_-enhanced radiation blocking is presented in Figure 2a [16]. Correspondingly, Figure 2b shows the schematic illustration of heat transfer pathways.

On the flame retardancy front, aerogels primarily function through a physical barrier mechanism, the efficacy of which is equally inseparable from their nanostructure. On one hand, inorganic aerogels (e.g., SiO_2_) possess inherent non-combustibility, providing a stable foundation for substrate protection. More significantly, however, their intact nanoporous structure can form a dense and stable physical barrier upon exposure to flame. This barrier effectively impedes the inward diffusion of external oxygen toward the substrate surface while simultaneously suppressing the outward release of combustible volatiles from within. Thereby, it physically disrupts the “fuel-oxygen” chain reaction, constituting the core physical mechanism behind the highly efficient flame retardancy of aerogels.

### 2.2. Synergistic Effects of Aerogels and Coatings

The synergistic effects between aerogels, flame retardants, and matrices are crucial for enhancing overall performance. This synergy, based on the porous structure of aerogels, enables the strengthening of multiple properties.

**Figure 3:**
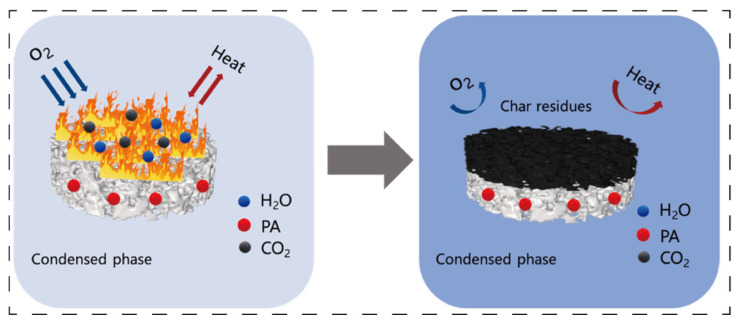
Phytic acid enhances the process of carbon layer formation [18].

In terms of synergy with flame retardants, the porous network of aerogels provides numerous loading sites for flame retardants, significantly improving their dispersibility and stability. When exposed to high temperatures, inert gases (such as H_3_PO_3_, NH_3_, and H_2_O) generated by the decomposition of flame retardants (e.g., phosphorus-based compounds, nitrogen-based compounds, aluminum hydroxide, and montmorillonite) [18,19,20,21] can be uniformly released through the pore structure of aerogels, effectively diluting the concentration of combustible gases in the combustion zone. Figure 3 exhibits the enhancement of phytic acid in the process of carbon layer formation [18]. Meanwhile, the aerogel skeleton can also delay the decomposition rate of flame retardants, extending their action time. The synergistic effect between the inorganic flame-retardant montmorillonite and aerogels is particularly prominent: the lamellar structure of montmorillonite and the aerogel network form a “nacre-like” hierarchical structure, which enhances high-temperature stability and reduces the transfer of heat and combustible gases. For example, the peak heat release rate of carboxymethyl chitosan/montmorillonite composite aerogels decreases by 58.4%, the char residue rate increases, the char layer becomes denser, and montmorillonite can catalyze the carbonization of the matrix, further strengthening the flame retardancy [22].

When combined with matrix materials, some fibrous or rod-like aerogels or those with specific structures can further reinforce the carbon layer structure [1,23]. They interact with the carbon layer formed during the combustion of the substrate: their nano-network skeletons can interpenetrate into the carbon layer, filling its pores and defects to form a denser and more stable composite carbon layer structure. This effectively blocks the transfer of heat to the interior of the substrate, thereby enhancing the structural stability of the material under high temperatures. Meanwhile, the matrix provides structural support for the aerogels, preventing them from breaking under stress or high temperatures and maintaining the integrity of their porous structure, which in turn ensures the stable performance of their thermal insulation and flame-retardant properties.

Overall, this synergistic effect is reflected in the composite barrier formed during combustion. The physical barrier of aerogels, the chemical inhibition of flame retardants, the structural support of the matrix, and the strengthened carbon layer structure under the joint action of the three work together to comprehensively block the combustion chain, significantly improving the overall fire safety and thermal stability of the material.

## 3. Characteristic Comparison and Selection Analysis of Different Aerogels

Following an understanding of the universal mechanism of aerogels, a subsequent crucial question arises—how to select the most suitable material from the diverse family of aerogels for fire-retardant coating formulations. The chemical nature and microstructure of aerogels fundamentally determine their thermophysical properties, mechanical behavior, and surface characteristics—intrinsic attributes that directly govern their suitability and final performance within coating systems. Consequently, systematic comparison and analysis of the properties of mainstream aerogels form the prerequisite for rational material design. This chapter will focus on four representative categories of aerogels—silica-based, carbon-based, bio-based, and metal oxides—to elaborate on their characteristic differences and selection criteria.

### 3.1. Silica-Based Aerogels

Silica-based aerogels are the most extensively researched and widely applied type in the field of fire-retardant coatings [9]. Their core advantage lies in an exceptionally low thermal conductivity (often below 20 mW·m^−1^·K^−1^ at ambient pressure) [16,24,25]. This originates from their nanoporous network, constructed from SiO_2_ nanoparticles with porosities exceeding 90%, which synergistically achieves highly efficient thermal insulation through convection suppression, phonon scattering, and radiation modulation. In terms of fire resistance, their primary component, silica, is intrinsically non-combustible and inorganic. However, the main challenges in their application are high brittleness and low mechanical strength, which can lead to coating cracking or powdering at high filler loadings [26]. Furthermore, the abundant surface silanol groups render them hydrophilic, necessitating hydrophobic modification to ensure stability within the coating system. Fortunately, these silanol groups also facilitate surface functionalization (e.g., using silane coupling agents), thereby improving the interfacial compatibility with the polymer matrix. From an engineering perspective, silica-based aerogels can already be produced as powders and slurries, offering good compatibility with existing coating application processes. Although their cost remains higher than that of traditional fillers, they currently represent the most viable option.

### 3.2. Metal Oxide Aerogels

Metal oxide aerogels (e.g., alumina—Al_2_O_3_ or zirconia—ZrO_2_) are regarded as potential solutions for surpassing the temperature limits of silica-based aerogels. Their most notable advantage is their exceptional high-temperature stability; many types can maintain structural integrity over prolonged periods at 1200 °C or even above 1500 °C, thus providing durable thermal protection in ultra-high-temperature fire scenarios [27,28,29,30].

Nevertheless, the path for these aerogels from the laboratory to the fire-retardant coatings market faces more severe challenges compared to their silica-based counterparts. Firstly, their nano-network skeletons are typically more mechanically fragile. This inherent structural weakness makes them more susceptible to nanopore collapse and sintering during subsequent crushing and processing, leading to the significant degradation of their core thermal insulation properties once powdered. Secondly, they commonly suffer from high-temperature phase transition issues (e.g., the γ→α transition in alumina during heating), a process often accompanied by substantial volume shrinkage and pore disappearance, causing non-linear, drastic changes in their insulating performance upon heating [28,31]. Finally, a decisive obstacle is their exceedingly high cost. This stems not only from expensive metal alkoxide precursors but also from their reliance on complex process control (such as incorporating additives to reinforce the skeleton) and even costly supercritical drying techniques.

Consequently, although metal oxide aerogels can technically be obtained in powder form through similar drying and crushing processes, their inherent structural fragility, phase transition issues, and prohibitively high cost make it extremely difficult to currently obtain a powder for fire-retardant coatings that is competitive in terms of cost-effectiveness with mature silica-based products. This confines metal oxide aerogels primarily to cost-insensitive applications in extreme environments, such as aerospace and defense, with large-scale adoption in the commercial fire-retardant coatings sector yet to be realized.

### 3.3. Carbon-Based Aerogels

Carbon-based aerogels (e.g., graphene and carbon nanotube aerogels) exhibit a distinctly different property profile to silica-based aerogels. They possess outstanding high-temperature resistance in inert atmospheres [32] and often demonstrate good elasticity and toughness, effectively avoiding issues of brittle fracture [33,34,35,36]. However, their most significant application barrier lies in chemical environmental stability: in an air atmosphere, oxidation begins at around 350 °C, with highly graphitized graphene or carbon nanotubes showing relative resistance up to approximately 570 °C [33], severely limiting their long-term reliability in open-flame fire scenarios. More critically, their macroscopic forms are mostly free-standing monoliths or films, making direct dispersion as fillers in coatings challenging. Therefore, the current research focus for carbon-based aerogels is primarily in areas like electromagnetic interference shielding and energy storage [32]. Their feasibility as functional fillers in fire-retardant coatings is very low, except potentially as auxiliary components for constructing conductive networks to enable functions like fire warning systems [37].

### 3.4. Bio-Based Aerogels

Bio-based aerogels (e.g., from sodium alginate, ammonium alginate, or cellulose) have attracted significant interest due to their renewable and biodegradable, environmentally friendly characteristics [18,38,39,40,41]. Their three-dimensional networks, composed of biomass macromolecules, often impart excellent flexibility and damage tolerance to the material, alongside low thermal conductivity. However, their intrinsic polyhydroxyl chemical structure results in strong hydrophilicity, making them prone to moisture absorption and structural collapse in humid environments, directly conflicting with the durability and water resistance required for coatings. The most fundamental challenge is their intrinsic combustibility; as organic materials, they themselves represent fuel rather than a fire retardant and must undergo profound fire-retardant modification before consideration for use in fire protection systems. Coupled with questions regarding their cost and long-term durability, bio-based aerogels currently remain largely within the realm of prospective basic research, representing a potential but as of yet unripe direction for future green fire-retardant coatings.

### 3.5. Comparative Analysis and Selection Perspective

In summary, the characteristic differences between the various types of aerogels dictate their distinct positions within the technology roadmap for fire-retardant coatings. For clarity, a comprehensive comparison of their key properties is provided in Table 1 below.

## 4. Technical Challenges in Applying Aerogels in Fire-Resistant Coatings

Having established an understanding of the mechanisms and properties of aerogels, the core challenges encountered during their integration into coating systems emerge as critical issues requiring clarification. This chapter will systematically dissect four major technical bottlenecks: the inherent conflict between nanopore preservation and interfacial compatibility; coating brittleness arising from the intrinsic mechanical weakness of aerogels; the conflict between high filler loading and coating workability; and the cost and process barriers to scalability. This systematic analysis aims to establish a clear problem-oriented framework for the subsequent discussion of solutions.

### 4.1. The Synergistic Dilemma of Nanopore Preservation and Interfacial Compatibility

The application of aerogels in fire-resistant coatings is fundamentally challenged by an inherent conflict rooted in their nanoporous structure: to maintain their ultra-low thermal conductivity, the nanoscale pores must be protected from infiltration or blockage by the polymer matrix. Yet, to ensure the coating’s mechanical integrity and long-term durability, strong interfacial bonding and the uniform, stable dispersion of the aerogel within the matrix are essential. This competition between opposing requirements constitutes the primary technical difficulty.

The most direct strategy for achieving the primary objective of pore protection involves the surface treatment of the aerogel to render its surface characteristics opposite to those of the liquid matrix. For instance, in water-based coating systems, hydrophobic surface modification is most commonly employed [43] to reduce the matrix’s wettability toward the pores. However, this critical measure directly creates an inherent compatibility mismatch between the aerogel and most polar polymer matrices. This poor compatibility simultaneously triggers two key technical problems: Firstly, the modified surface struggles to form effective chemical bonding and physical anchoring with the resin matrix, severely weakening the interfacial adhesion and leading to diminished coating adhesion and overall mechanical performance. For example, the bonding strength of a high-temperature coating—prepared by incorporating a hydrophobic SiO_2_ aerogel into an aqueous silica sol—to a wall putty substrate was only 0.2 MPa [44], not meeting the industry standard of 0.3 MPa [45]. Secondly, the inherent incompatibility makes it challenging to achieve uniform and stable dispersion during processing.

Consequently, a technological breakthrough for aerogels in fire-resistant coatings largely depends on finding the optimal balance point—through meticulous surface design and process control—between the conflicting demands of “pore protection” and “interfacial bonding/dispersion stability”. Specific strategies and recent advances for addressing these compatibility challenges are detailed in the following section.

### 4.2. Insufficient Intrinsic Mechanical Properties and Brittleness of Composite Coatings

As outlined in Section 3, brittleness is a universal intrinsic property of aerogels. However, when incorporated as fillers in fire-resistant coatings, this characteristic triggers a cascade of adverse effects, posing a significant application challenge.

Firstly, the low strength and high friability of aerogels make them highly susceptible to fracture during high-speed dispersion, grinding, and application processes (e.g., the impact during spraying). This not only causes the collapse of nanopores—leading to a permanent degradation of thermal insulation performance—but also generates a substantial number of fine fragments. These fragments increase the system viscosity and adversely affect application rheology.

Furthermore, once introduced into the coating, these brittle particles act as internal stress concentration points. Under external stress (such as substrate deformation or impact) or thermal stress (e.g., from thermal cycling), microcracks readily initiate and propagate from these weak points. This results in a marked decline in coating flexibility, impact resistance, and adhesion. The direct consequence is that the coating becomes prone to developing microcracks or even delamination during service. Once the coating’s integrity is compromised, its function as a fire and thermal barrier is instantly lost, allowing flames and heat to penetrate unimpeded and causing the entire fire protection system to fail.

Moreover, the mechanical shortcomings arising from this brittleness severely constrain the maximum effective loading level of the aerogel in the coating. To maintain the most fundamental structural integrity and adhesion, formulators are often compelled to sacrifice the higher aerogel content, thereby compromising the coating’s theoretical thermal insulation limit. Consequently, the brittleness of aerogels is not merely a material property drawback, but a critical bottleneck hindering their application in high-performance fire-resistant coatings.

### 4.3. The Balance Contradiction Between High Filler Content and Comprehensive Performance

A fundamental conflict exists between the high thermal insulation performance offered by aerogels and the concomitant deterioration in the coating’s mechanical properties, workability, and durability. These competing factors are mutually restrictive, directly limiting the feasible aerogel loading ratio and suitable application scenarios. As the aerogel content increases, while the coating rigidity may see a marginal improvement due to a perceived “skeletal support” effect, its flexibility and adhesion are significantly compromised [26]. This renders the coating prone to cracking and delamination under the thermal expansion/contraction of the substrate or external impact. Crucially, any breach of the coating allows flames and heat to directly attack the substrate, resulting in the complete failure of the fireproof barrier.

Concurrently, the high aerogel loading substantially increases the coating’s viscosity, attributable to the nanoporous structure and high specific surface area. This leads to application issues such as difficult atomization and poor leveling. Attempting to mitigate this via dilution can destabilize the dispersion, causing the uneven distribution of the insulating filler and creating localized “fireproofing blind spots”.

Furthermore, the nanoporous structure of aerogels is inherently more susceptible to environmental degradation. At high loadings, a greater volume of these sensitive nanopores is exposed. In outdoor or humid environments, moisture ingress can cause pore collapse, while ultraviolet radiation accelerates the degradation of surface functional groups. This not only leads to coating powdering and flaking but also destroys the nanopore-driven thermal insulation mechanism, causing a rise in thermal conductivity. Consequently, the high-efficiency insulation initially provided by the aerogel is gradually lost, ultimately failing to maintain the designed fire resistance rating.

These conflicts represent an intrinsic clash between the requirement for a high aerogel content and the practical performance needs of a functional coating. This makes it exceptionally difficult to find an optimal balance during performance optimization and practical applications, presenting a prominent obstacle in the industrialization process.

### 4.4. Barriers of Scalable Production: Cost and Technical Compatibility

The transition of aerogels from laboratory research to industrial-scale production necessitates overcoming inherent conflicts between their synthesis protocols and the requirements of mass manufacturing. These conflicts, primarily reflected in prohibitively high costs and process bottlenecks incompatible with continuous production, constitute the core obstacles to their large-scale application in fire-resistant coatings.

On the cost front, the combined “material-process” expense structure of aerogels renders their price substantially higher (dozens to hundreds of times) than that of conventional fillers [46,47]. Regarding raw materials, high-performance aerogels often rely on costly high-purity precursors (e.g., tetraethyl orthosilicate, TEOS) or specialized chemical agents. Process wise, the decisive drying stage represents the major cost center. While supercritical drying can perfectly preserve the nanostructure, its enormous capital investment, high energy consumption, and operational safety costs directly result in elevated product prices, severely limiting the economies of scale. Ambient pressure drying, as the most promising alternative, significantly reduces the equipment costs but relies on complex pre-treatment steps (e.g., multi-step solvent exchange and surface modification) to counteract the shrinkage and cracking caused by capillary forces, thereby increasing the process complexity and time expenditure. Although freeze-drying is applicable for some organic aerogels, its equipment and energy costs remain higher than those of ambient pressure drying, and its lengthy process cycle constricts production efficiency.

On the process front, a fundamental mismatch exists between the “batch-based, long-cycle” logic of aerogel production [47] and the “continuous, high-efficiency” workflow of the coatings industry. The aerogel synthesis cycle—from gelation and aging to drying—spans several days, incompatible with the hourly batch rhythm of a coatings production line. This forces the insertion of an independent, capital-intensive aerogel pre-processing stage into the supply chain. A more severe challenge during production scale-up is that the intense shear forces generated by high-speed dispersing equipment readily cause the fragmentation of brittle aerogel nanoparticles and pore collapse. This leads to the irreversible degradation of insulation performance, batch-to-batch inconsistency, and introduces safety risks such as dust explosion, presenting formidable challenges for industrial continuous production.

## 5. Composite Modification Strategies of Aerogels in Fire-Retardant Coatings

Building upon the identified application challenges, this section will systematically elaborate on targeted composite modification strategies. Specifically, it will sequentially examine technical pathways for resolving interfacial compatibility, enhance mechanical properties, balance coating workability, and achieve low-cost scalability—thereby providing a clear research and development framework for constructing high-performance aerogel-based fire-retardant coating systems.

### 5.1. Regulation of Compatibility with Resin Matrices

The nanoporous structure of aerogels creates significant disparities in surface energy and density compared to coating matrices, frequently causing dispersion inhomogeneity or agglomeration due to interfacial tension mismatch. This consequently compromises the mechanical properties and fire stability of composites. Therefore, compatibility regulation constitutes the primary step in composite modification. Achieving this requires coordinated breakthroughs across three dimensions: surface chemical modification, interfacial dispersion regulation, and dispersion process optimization.

#### 5.1.1. Surface Chemical Modification

Surface chemical modification serves as the key approach for enhancing compatibility in composite systems. By precisely regulating the polarity, reactivity, and spatial configuration of surface functional groups in aerogels, it effectively reduces interfacial energy disparities with the coating matrix while establishing stable chemical bridges (e.g., covalent bonds, hydrogen bonds, or coordination bonds). Current modification pathways primarily include hydrophobic modification, hydrophilic modification, and silane coupling agent modification. The selection of these techniques requires rigorous alignment with specific coating system characteristics and application requirements, demonstrating significant scenario dependency.

**Figure 4:**
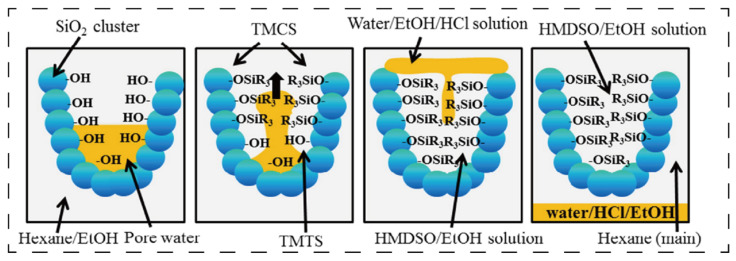
Schematic diagram of the surface modification process using the TMCS/hexane/EtOH agent [48].

Hydrophobic modification represents the most prevalent approach for commercial SiO_2_ aerogels. This preference stems from the inherent hydrophilicity of abundant surface silanol (-SiOH) groups on pristine aerogels, combined with their extremely high specific surface area, which readily adsorbs atmospheric moisture, compromising structural integrity. In practice, alkylation reagents such as trimethylchlorosilane (TMCS) and hexamethyldisilazane (HMDS) replace surface hydroxyl groups with hydrophobic moieties (e.g., -CH_3_). This treatment elevates the water contact angles beyond 120°, significantly enhancing compatibility with non-polar resins. Figure 4 shows the schematic diagram of the hydrophobic modification of SiO_2_ aerogels using hexane/ethanol/trimethylchlorosilane (hexane/ethanol/TMCS) [48]. Consequently, hydrophobic aerogels are widely adopted in epoxy resin and silicone resin systems [49,50,51].

In contrast, hydrophilic modification—achieved by retaining or introducing polar groups (e.g., carboxyl and amino)—proves more suitable for aqueous resin systems. Within water-based acrylic systems, commercially available hydrophobic SiO_2_ aerogels typically undergo secondary hydrophilic modification prior to incorporation into coatings. Such dual-modified aerogels preserve the intrinsic hydrophobicity within the particle core while enhancing interfacial bonding with aqueous coatings via external hydrophilic groups, enabling homogeneous dispersion at the nanoscale [52,53].

Silane coupling agent (SCA) modification demonstrates superior versatility, where reactive groups at both molecular termini (e.g., alkoxy vs. amino/epoxy) react with hydroxyl groups on aerogel surfaces and functional groups of resins, respectively, forming “molecular bridges” at the interface. For instance, SCA-modified SiO_2_ aerogels exhibit enhanced performance in cement mortar systems, with freeze-thaw resistance showing significant improvement [43,54].

Table 2 summarizes the data on selected aerogel surface modification strategies and their influence on the properties of composite materials. It can be observed that dual-modified SiO_2_ aerogels show clear evidence of a protected porous structure in acrylic resin systems [52], while silane coupling agent-modified aerogels exhibit particularly outstanding performance in cement mortar matrices [43,54]. Furthermore, the current literature reveals that the impregnation behavior of aerogels has not been thoroughly investigated. Many studies lack comparative analysis of mechanical or thermal insulation properties post modification, and the most direct microstructural characterization remains insufficiently precise. Notably, a significant disparity exists in research depth across different resin systems: beyond the epoxy, silicone, and waterborne acrylic resins mentioned, surface modification studies in polyurethane, phenolic, and amino resin matrices remain relatively scarce.

#### 5.1.2. Surfactant-Assisted Modification

Distinct from the robust interfacial bonding achieved through the surface chemical modification of solid-phase functional groups, surfactant-assisted modification utilizes hydrophilic and hydrophobic moieties within molecular structures to reduce interfacial tension and enhance liquid spreading on solid surfaces. Concurrently, it ensures long-term dispersion stability via electrostatic repulsion or steric hindrance effects. This approach specifically improves the wettability and dispersion stability of aerogels within resin matrices of varying polarities.

In aqueous polyurethane systems, the wetting agent H1400 reduces the solid–liquid interfacial tension, facilitating emulsion wetting on particle surfaces and strengthening interfacial bonding between inorganic fillers and organic matrices. This significantly enhances the overall coating uniformity and stability [59]. For water-based acrylate systems, dispersants such as sodium dodecyl sulfate (SDS) stabilize aerogel dispersions through steric hindrance, further optimizing compatibility [60]. In epoxy resin systems, the wetting agent BYK-P 9920 accelerates surface spreading by reducing the resin’s surface tension, thereby minimizing the interfacial defects on aerogel particles [61].

#### 5.1.3. Optimization of Dispersion Processes

Physical dispersion processes are effective methods for inhibiting aerogel agglomeration. Among them, the ultrasonic dispersion process generates cavitation effects in low-viscosity solvents through high-frequency mechanical vibration, impacting and dispersing aerogel agglomerates. For example, when graphene aerogels are compounded with epoxy resins, ultrasonic mixing can effectively exfoliate graphene sheets and disperse them uniformly in the resin matrix [62]. The in situ polymerization method achieves uniform distribution with the growth of molecular chains during polymerization by pre-dispersing aerogels in resin monomers, which can avoid the secondary agglomeration caused by mechanical mixing. For instance, by introducing pre-treated silicon-based aerogels into the synthesis of polyethylene terephthalate, the dispersion uniformity of aerogels in the matrix is significantly improved after in situ polymerization, and the thermal conductivity of the composite material is significantly reduced [63].

### 5.2. Mechanical Enhancement and Integrated Structure–Function Design

Addressing the inherent brittleness of aerogels and the consequent mechanical degradation of composite coatings, this section focuses on design strategies for mechanical reinforcement and functional integration. It encompasses multiple approaches—from fiber reinforcement and polymer cross-linking to bio-inspired structural design—emphasizing how these methods enhance the strength and toughness of the aerogel material itself while preserving its fire-retardant and thermal insulation functions.

#### 5.2.1. Construction of Oriented Structures

**Figure 5:**
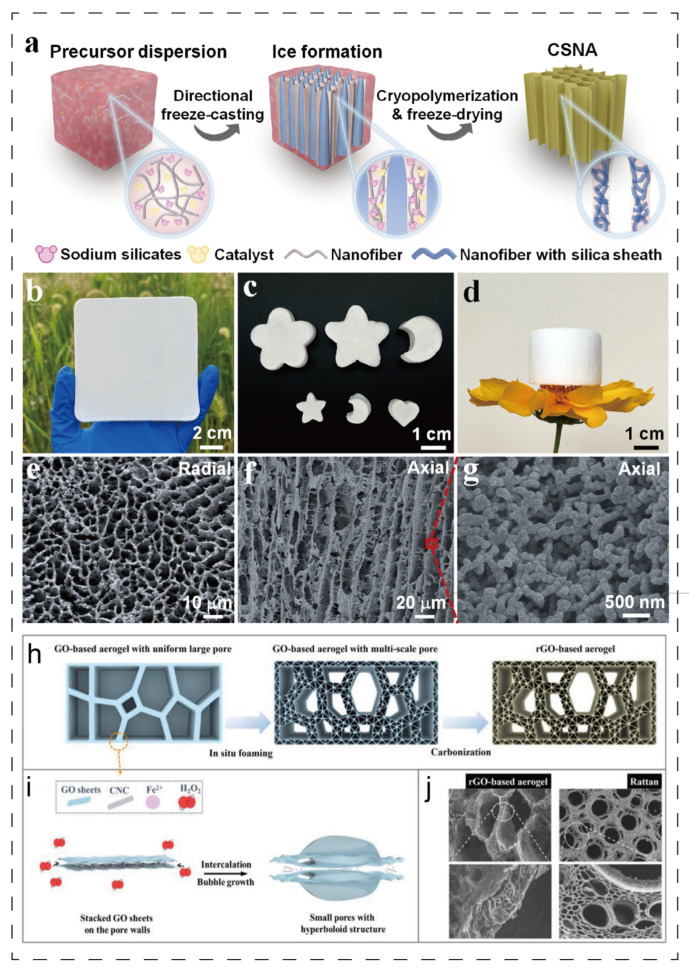
(**a**) Schematic displaying the cryopolymerization procedures of the CSNA featuring silica-sheathing nanofibrous architectures; (**b**–**d**) Photographs showing the CSNA in a large size, in diverse shapes, and standing on a flower, respectively; (**e**–**g**) SEM image of CSNA perpendicular to the freezing direction and along the freezing direction, revealing the interwoven nanofibrous network [64]; (**h**) Schematic illustration of the fabrication of “rattan-like” aerogel; (**i**) The formation of small pores; and (**j**) SEM images of the obtained aerogel and the rattan [65].

The microstructural orientation of aerogels can be engineered through techniques such as directional freeze-casting and magnetic field-induced alignment, thereby conferring anisotropic thermal insulation and mechanical properties upon the composite coatings. Specifically, directional freeze-casting utilizes a temperature gradient to drive the oriented growth of ice crystals. This process compels the aerogel framework—comprising components such as graphene sheets or nanofibers—to align along the ice growth direction, resulting in highly ordered lamellar or columnar architectures. Such aligned structures significantly enhance material performance along specific orientations [36,64,65,66]; Figure 5a–g present a composite aerogel comprising nano-fibrillated cellulose and SiO_2_ fabricated via directional freezing technology. Figure 5a provides a schematic of the preparation process, while Figure 5b–d display optical images at different scales. The corresponding microstructure of the aerogel is shown in Figure 5e–g. Its aligned hierarchical porous structure confers remarkable mechanical elasticity and thermal superinsulation properties [64]. Separately, Figure 5f–h illustrate a vine-like graphene-based composite aerogel also produced via directional freezing, with Figure 5f–i depicting its fabrication schematic and Figure 5h revealing its microstructure. This unique porous architecture, featuring a dual-connected network, endows the aerogel with excellent mechanical stability [65].

**Figure 6:**
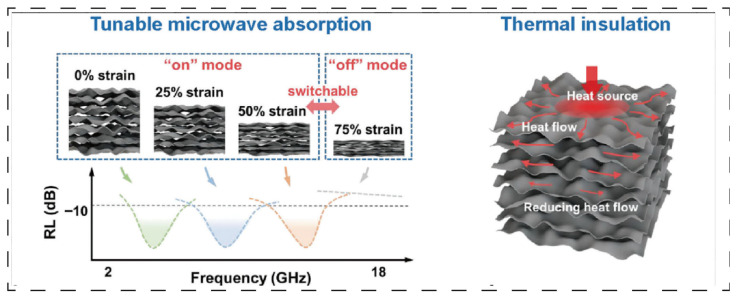
Schematic illustration of carbon spring aerogel in tunable microwave absorption performance and thermal insulation capability, respectively [36].

Figure 6 depicts a carbon spring aerogel (FCS) [36] whose unique long-range layered multi-arch microstructure and anisotropic thermal conductivity are achieved through freeze-forming technology combined with additional processing. This design enables the synergistic optimization of directional scattering and absorption of thermal radiation, yielding an exceptionally low thermal conductivity of 12.7 mW·m^−1^·K^−1^ in the vertical direction and demonstrating superior thermal protection performance. The material additionally exhibits tunable microwave absorption properties. Furthermore, by precisely tailoring the pore size distribution of aerogels—such as through hierarchical pore structure design—the suppression of heat radiation and convection can be further enhanced, improving the high-temperature thermal insulation stability by over 20%. This structural enhancement mechanism demonstrates universal applicability, as evidenced by laminated aramid nanofiber aerogels that increase the thermal decomposition temperature of composites from 200 °C to 250 °C while significantly increasing the char residue [67]. For further reference, Table 3 summarizes additional examples of aerogels with aligned structures beyond those cited here.

#### 5.2.2. Bionic Structure Design

**Figure 7:**
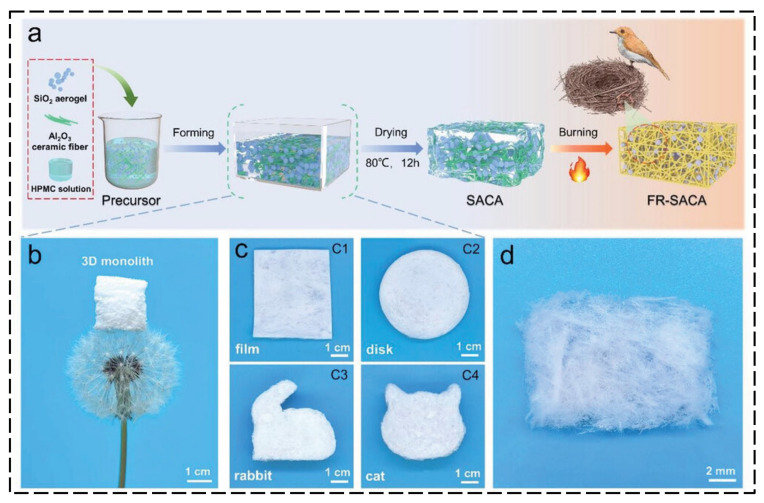
Imitation bird’s nest structure FR-SACA [69]. (**a**) Schematic illustration of the manufacturing process of SACAs and FRSACAs; (**b**) Image of a SACA placed on top of a dandelion; (**c**) Optical photographs of SACAs with various shapes; and (**d**) Optical photograph of an FR-SACA.

By simulating biological structures in nature with excellent mechanical and thermal insulation properties (such as a bird’s nest structure, honeycomb structure, shell layered structure, and cell layered structure), the skeleton strength and heat barrier efficiency of aerogels are optimized. For example, the bird’s nest–mimetic structure draws on the branch-like interwoven network of bird nests, using nanofibers (such as cellulose nanofibers and carbon nanofibers) as “nest branches” and aerogel particles as “nest fillers”. A three-dimensional network is formed through the disordered interweaving of fibers, and aerogel particles are embedded in the gaps and bonded with fibers. This structure increases the impact strength of composite coatings by more than 50% through the “bridging-energy dissipation” effect, and the multi-level pores (micropores between fibers and nanopores of aerogels) synergistically inhibit heat transfer, reducing the thermal conductivity to below 60 mW·m^−1^·K^−1^. At high temperatures, the fiber skeleton can prevent the collapse of aerogel pores, significantly enhancing the thermal insulation stability [67,68]. Figure 7 presents a bird’s nest-inspired composite aerogel (SACA) integrating silica aerogel microparticles with Al_2_O_3_ ceramic fibers [69]. Figure 7a illustrates the synthesis procedure, while Figure 7b–d depict its macroscopic morphologies. The SACA demonstrates exceptional properties, including an ultra-low density of 10 mg cm^−3^, a thermal conductivity of 29 mW·m^−1^·K^−1^, and a remarkable reversible compressibility of up to 80%.

Honeycomb–mimetic aerogels [1,70], through the ordered arrangement of hexagonal units, can increase the compressive strength of composite coatings by 30–50% under the same porosity, and the inhibition effect of hexagonal units on air convection can further reduce the thermal conductivity by 10–15%; shell–mimetic “brick-mortar” structures (aerogels as “bricks” and resin/flame retardant composites as “mortar”) [71] can improve the impact resistance of coatings through interface synergistic effects (impact strength ≥ 5 kJ/m^2^), and the interlayer dislocation structure can extend the heat transfer path, increasing the thermal insulation stability at high temperatures by more than 25%. By emulating the layered structure of leaves—comprising the epidermis, mesophyll, and vascular bundles [72]—the silica/chitosan/zirconia fiber composite aerogel (SCZ) is endowed with exceptional characteristics including low density, mechanical robustness, high-temperature dimensional stability, and low thermal conductivity. These properties are achieved through the effective obstruction of heat transfer and prevention of structural collapse under localized stress. For further reference, Table 3 summarizes additional examples of bio-inspired aerogel structures beyond those cited here. Such biomimetic structural designs provide novel pathways for reconciling the inherent lightweight nature of aerogels with the mechanical reliability required in coating applications.

#### 5.2.3. Fiber Reinforcement

The incorporation of fibers (e.g., carbon fiber, quartz fiber, ZrO_2_ ceramic fiber, and basalt fiber) compensates for aerogel brittleness through a “bridging-toughening” effect while synergistically enhancing the mechanical properties and fire stability of coatings. Specifically, fibers span interfacial cracks between aerogels and resin matrices, dissipating energy via tensile deformation and interfacial friction—doubling the coating strength. For instance, ZrO_2_ fibers increase the compressive strength of ZrO_2_-SiO_2_ aerogel composites from 0.36 MPa to 0.82 MPa [73], short-cut carbon fiber-reinforced silica-phenolic resin (Si/PR) aerogel nanocomposites show a strength elevation from 0.33 MPa to 2.44 MPa [74], and needle-punched quartz fiber composites with multi-walled carbon nanotube-modified silicone resin aerogels achieve thermal conductivity of 54–75 mW·m^−1^·K^−1^ [75]. Concurrently, high-temperature-resistant fibers (e.g., basalt fiber, >800 °C tolerance) act as skeletal reinforcements during fires, inhibiting the high-temperature collapse of aerogel structures and extending the effective duration of thermal barriers [76].

#### 5.2.4. Multi-Component Collaboration

Beyond fibers, compounding aerogels with other functional fillers (e.g., carbon nanotubes, graphene, or flame retardants) enables the construction of multi-component “aerogel-fiber-nanofiller” systems, achieving the synergistic enhancement of fire resistance, mechanical properties, and durability. For instance, a graphene aerogel self-assembled on carbon fibers improves the interfacial performance with epoxy resin [77]. The interconnected graphene aerogel network acts as a bridge, ensuring efficient stress transfer from epoxy to carbon fibers and elevating the interfacial shear strength from 38.8 MPa to 71.1 MPa. Silicone resin composites incorporating halloysite nanotubes (adsorbed with phosphorus-containing flame retardants) and silica aerogels exhibit significantly enhanced thermal insulation, smoke suppression, and flame retardancy through component synergy [50]. The organic–inorganic dual-network architecture—exemplified by the phenolic resin/SiO_2_ system—leverages a continuous nanoscale interpenetrating network to retain the processability inherent to the organic component, while capitalizing on the thermal stability of the inorganic framework under high temperatures. This synergistic combination delivers exceptional thermal insulation performance, maintaining a backside temperature of merely 300 °C when exposed to a 1300 °C flame [78]. The self-interlocked 3D skeleton of the Ti_3_C_2_T_x_;/polyvinyl alcohol (PVA) aerogel reduces toxic gas emissions and enhances the char layer stability, substantially improving flame retardancy, impact resistance, and electrical conductivity [79]. These multi-component aerogel composite strategies provide comprehensive performance assurance for complex fire scenarios.

**Table 3:** Effects of aerogel mechanical enhancement strategies on the performance of aerogels.

Strategy	Aerogel Composition	Structure Characteristics	Density mg cm^−3^	Mechanical Properties	Thermal Conductivity mW·m^−1^·K^−1^	Ref.
Oriented structure	C–Graphene–BNNS	Long-range layered multi-arch structure	9.3–18.7	Reversible compressive strain within 75%	12.7 vertical 70.4 parallel	[36]
	SiO_2_ + nanocellulose	Honeycomb layered anisotropic microporous–SiO2 micropores	55	Reversible compressive strain within 70%	23 vertical 93 parallel	[64]
	Graphene–carbonized nanocellulose	Rattan macropores–foamed pores	11.7	200 strain cycles within 60%	/	[65]
	Graphene	Parallel cylindrical holes	0.23–5.1	/	/	[66]
Bionic structure	ZrO_2_–Al_2_O_3_–mullite fiber	Imitation bird’s nest multi-level holes	450	Compressive strength: 1.05 MPa (10 times pure aerogel)	52.4	[68]
	SiO_2_–Al_2_O_3_ fiber	Imitation bird’s nest multi-level holes	10	Reversible compressive strain within 80%	29	[69]
	SiOC	Imitation of cellular structure	87	Compressive strength: 0.3–0.34 MPa	51–57	[70]
	SiO_2_–cellulose nanofibers–hydroxide	Imitation brick–mud structure	22.1	Reversible compressive strain within 80%	29.6	[71]
	SiO_2_–chitosan–ZrO_2_ fiber	Leaf-like structure	51	Reversible compressive strain within 80%	30	[72]
Fiber reinforcement	SiO_2_–ZrO_2_ fiber	Fiber uniform distribution	290	Compressive strength: 0.82 vs. 0.36 MPa (fiber-free)	23.5–29.6	[73]
	Carbon fiber–silicone phenolic resin	Fiber uniform distribution	402–463	Compressive strength: 2.44 vs. 0.33 MPa (fiber-free)	92–119	[74]
	Quartz fiber–CNT–silicon resin	Fiber uniform distribution	372–427	/	54–75	[75]
	SiO_2_–basalt fiber	Fiber uniform distribution	297–321	Compressive strength: 0.93 vs. 0.18 MPa (fiber-free)	26.3–36.2	[80]
	SiO_2_–Al_2_O_3_–mullite fiber	Fiber uniform distribution	380–390	Compressive strength: 0.1–0.13 MPa	40	[76]
	Graphene–Cf–epoxy resin	Fiber uniform distribution	/	Shear strength: 71.1 vs. 38.8 MPa (fiber-free)	/	[77]

### 5.3. Strategies for Balancing Coating Workability and Functional Performance

To realize the practical application of aerogel-based fire-resistant coatings, a balance must be achieved between high thermal insulation on the one hand, and applicability (workability) and mechanical performance on the other. This subsection systematically discusses strategies—including content optimization, particle grading, and rheological modification—to synergistically enhance the overall application performance of the coatings.

#### 5.3.1. Aerogel Volume Fraction Upper Limit and Optimal Content Window

In coating systems, the addition level of aerogel is inherently a matter of regulating its volume fraction, not simply its mass proportion. Due to their significantly lower density (~0.1 g/cm^3^) compared to the resin matrix (>1.0 g/cm^3^), aerogels can occupy a substantial volume within the composite coating system even at low mass fractions. Research indicates that at a mass fraction of 8%, the volume fraction may already approach 50%, meaning that the aerogel solid phase occupies nearly half the physical space of the mixed system.

This high-volume fraction state directly confronts the physical upper limit described using packing theory. For irregularly shaped, high-surface-energy aerogel particles, their actual packing state in the resin more closely resembles random loose packing, whose theoretical maximum volume fraction typically does not exceed 64%. When the aerogel volume fraction approaches or exceeds this critical value, the resin matrix struggles to adequately encapsulate each particle, leading to significant interfacial defects within the system. These defects manifest initially as direct particle-to-particle contact, creating stress concentration points that markedly reduce the coating’s flexibility and adhesion. This subsequently triggers a comprehensive deterioration in application properties: increased internal frictional resistance causes an exponential rise in coating viscosity, loss of leveling leading to application difficulties, and critically, due to agglomeration and interfacial thermal bridging, the thermal insulation improvement from excessive aerogel addition exhibits a trend of marginal diminishing returns.

Consequently, the core of content gradient optimization lies in identifying a rational “performance-processability” balance window. Synthesizing the literature reports, for most resin systems, the optimal aerogel volume fraction window typically lies within 50% (corresponding to ~8 wt%) [49,50,51,60]. Within this optimized range, the aerogel can be uniformly dispersed, while the resin matrix retains a sufficient continuous phase to encapsulate particles and transfer stress, thereby enabling the final coating to possess satisfactory thermal insulation, reliable mechanical properties, and acceptable application viscosity.

#### 5.3.2. Particle Grading and Rheological Control

Optimizing the content alone is insufficient to fully address the workability challenges posed by high additive loads. Therefore, particle grading is widely employed as an effective auxiliary method. By blending aerogel powders of different particle sizes (e.g., micron-scale and nanoscale), smaller particles can fill the voids between larger ones. This strategy increases the packing density of the filler without significantly raising the system’s porosity, thereby reducing the coating viscosity at the same solid content and improving leveling and the application experience.

Simultaneously, efficient rheological modifiers represent another key element for balancing workability. Introducing an appropriate number of thixotropic agents (such as fumed silica or organic bentonite) imparts desirable thixotropy to the coating: viscosity decreases under the application of shear stress, facilitating spraying or brushing, and rapidly recovers once the application ceases, ensuring coating uniformity and consistent final fire protection performance.

#### 5.3.3. Synergistic Enhancement of Processability via Surface Modification

As outlined in Section 5.1, the primary goal of the surface hydrophilization of aerogels is to protect the nanoporous structure. However, this modification concurrently delivers a synergistic benefit for improved processability: the hydrophobic treatment effectively reduces the cohesiveness and hygroscopicity between aerogel particles, thereby diminishing their tendency to agglomerate during storage and mixing. This inherently creates favorable conditions for producing high-solid-content, low-viscosity uniform pastes, indirectly supporting the objective of workability optimization.

### 5.4. Technical Pathways for Low-Cost and Scalable Production

To overcome the economic and process compatibility barriers hindering the transition of aerogels from laboratory research to industrial application, technological development is advancing along three main fronts: the utilization of low-cost raw materials, process intensification, and the adaptation of product forms. These strategies collectively aim to reduce the overall application cost of aerogels and facilitate their seamless integration into existing industrial coating production chains.

At the source of cost reduction, developing and applying low-cost precursors is pivotal. The current research and preliminary industrial practices demonstrate that using inexpensive sodium silicate [51,81] or rice husk ash [49] as silicon sources—replacing high-purity alkoxysilanes like tetraethyl orthosilicate (TEOS)—can substantially lower the raw material costs. Although the sodium silicate system presents greater challenges in gel control and impurity removal, process optimizations such as ion exchange and precise pH adjustment now enable the production of silica aerogels with properties suitable for fire-resistant coatings. This represents the most promising viable pathway for reducing material costs.

Concurrently, innovation in drying technology is central to lowering energy consumption and capital investment. Ambient pressure drying, aimed at replacing the expensive and safety-critical supercritical drying technique, has emerged as the dominant direction for scalable production [82]. This approach employs a series of meticulous pre-treatment strategies—such as network strengthening during gel aging and the use of low-surface-tension solvents combined with gel surface hydrophobic modification during solvent exchange—thereby significantly mitigating the destruction of the nanoporous structure via capillary forces during drying. It enables the direct production of aerogels with a high specific surface area and low thermal conductivity under ambient pressure, paving the way for large-scale, low-cost manufacturing.

Addressing compatibility with coating production processes necessitates the development of specialized aerogel pre-treatment and composite technologies. To tackle the issues of aerogel powder fragility and difficult dispersion during high-speed coating mixing, a proven effective method involves pre-preparing a high-solid-content, stabilized aerogel pre-dispersion paste by combining the aerogel with a portion of the resin and wetting/dispersing agents [83,84]. This ‘paste-based’ approach not only protects the aerogel structure and simplifies the coating production process but, crucially, allows the aerogel to be treated as a standardized liquid raw material. This facilitates seamless integration into existing coating production workflows for liquid ingredient metering and mixing, resolving the challenge of continuity in scaled-up production.

In summary, a clear and viable technical pathway for the scaling and cost reduction of aerogel-based fire-resistant coatings constitutes combining low-cost silicon sources with ambient pressure drying to reduce unit costs, complemented by product form innovation via pre-dispersion pastes to solve process compatibility issues. As these technologies mature and gain wider adoption, the economic and technical barriers to the application of aerogels in fire-resistant coatings are expected to be significantly overcome.

## 6. Application Fields and Research Status

**Figure 8:**
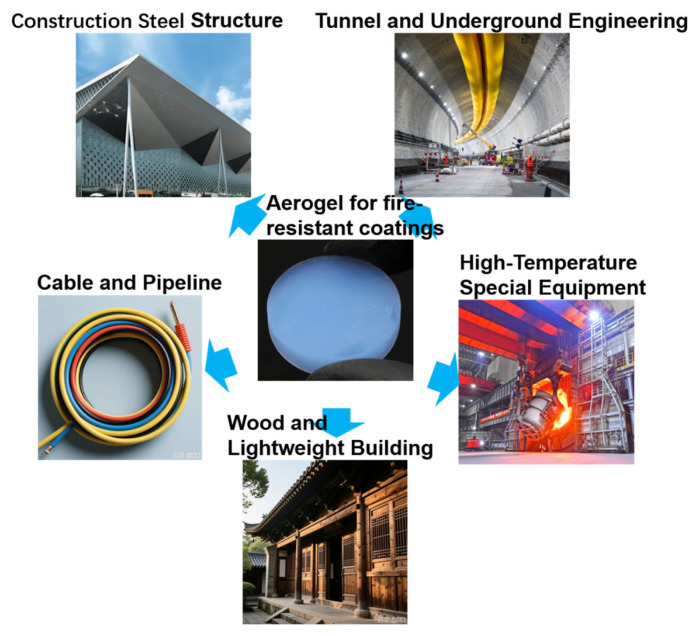
Application fields of fire-resistant coatings reinforced by the aerogel.

Among aerogel material systems, carbon-based aerogels (carbon aerogels and graphene aerogels), organic aerogels, and biomass aerogels exhibit unique advantages such as high conductivity, high mechanical elasticity, and excellent electromagnetic shielding performance. In the field of fire protection, they can realize functions like intelligent early warning through component forms (e.g., fire monitoring components based on conductive properties) [34,35,37]. However, their application forms are fundamentally different from the fire-retardant coatings focused on in this paper. The core advantages of fire-retardant coatings lie in easy constructability (e.g., simple processes such as brushing and spraying), universal fire protection applicability (adaptable to various substrate surfaces), and engineering practicality (capable of large-scale application in construction and transportation, etc.). In contrast, the component-based applications of the aforementioned aerogels require complex design and development processes and are not easily compatible with large-scale coating construction scenarios. Additionally, such aerogels have not yet broken through key engineering indicators such as industrialization, cost control (e.g., the high price of graphene aerogels), and long-term stability (organic aerogels are prone to degradation in humid environments), resulting in a significant gap in the practical application requirements of coating systems. Therefore, this chapter will not delve into such aerogels but focus on inorganic aerogels, which are more mature in research and more compatible with the characteristics of fire-retardant coatings, along with their applications. Figure 8 exhibits some application fields.

### 6.1. Construction Steel Structure Field

Construction steel structures are an important application scenario for fire-retardant coatings. In the event of a fire, fire-retardant coatings reduce the heating rate of steel through their own physical or chemical effects (e.g., thermal insulation, heat absorption, or intumescent flame retardancy), preventing steel from losing its load-bearing capacity due to high temperatures in a short time (the strength of steel decreases to less than 50% of that at room temperature, around 500 °C). Strict requirements are imposed on the coating’s thermal insulation performance, fire resistance rating, and adhesion to steel structures. Inorganic aerogels play a key role in this field by improving the high-temperature stability and thermal insulation efficiency of coatings.

Based on traditional steel structure fire-retardant coatings, the introduction of silica aerogels can significantly optimize the fire resistance of steel structure fire-retardant coatings. For example, in intumescent fire-retardant coatings, silica aerogels compounded with epoxy resin can reduce the cell size of the intumescent char layer and increase the expansion ratio to 18.3. Under a gas lamp flame for 1 h, the back surface temperature is only 260 °C, significantly enhancing the fire and thermal insulation performance of epoxy-based fire-retardant coatings [85]. Silica aerogels compounded with mullite fibers form ultra-thin intumescent fire-retardant coatings, which can form a dense and uniform “honeycomb” char layer, controlling the maximum back temperature of the steel plate at 337.8 °C, further verifying their excellent performance in steel structure fire protection [86]. In gypsum-based fire-retardant coatings, the incorporation of SiO_2_ aerogels with internal hydrophobicity and external hydrophilicity can improve the interface bonding between aerogels and the gypsum matrix, thereby significantly enhancing the lightweight property and fire resistance of the fire-retardant coating, with a fire resistance time of 112 min, far exceeding the conventional requirements [87]. In silicone resin-based fire-retardant coatings, the addition of SiO_2_ aerogels reinforced with halloysite nanotubes can greatly reduce smoke production, with a total smoke release of only 3.9 m^2^, effectively meeting the flame retardancy requirements of silicone resins in construction applications [50]. In fire-retardant and anti-corrosive coatings, SiO_2_ aerogels are compounded with organic montmorillonite and Al(OH)_3_/Mg(OH)_2_ to prepare water-based multi-functional coatings, which can achieve controlled thermal insulation and excellent corrosion resistance. They maintain a high corrosion inhibition efficiency (99.9%) in 3.5% NaCl solution, making them suitable for coastal steel structures [21].

### 6.2. Tunnel and Underground Engineering Field

Fire-retardant coatings for tunnels and underground engineering, due to the enclosed environment, narrow space, rapid smoke diffusion during fires, sudden temperature rise, and high rescue difficulty, have much higher requirements for fire protection than ordinary buildings. They need to withstand high-temperature smoke scouring, mechanical vibration, and humid environments, thus imposing high demands on the environmental friendliness of components, high-temperature resistance, water resistance, and lightweight property of fire-retardant coatings. The mainstream fire-retardant coatings are cement-based non-intumescent fire-retardant coatings, with internal fillers mostly being inorganic non-combustible components. Inorganic aerogels adapt to this scenario by optimizing the structural porosity, durability of coatings, and their own non-combustibility.

Silica aerogels have successful applications in cement-based tunnel fire-retardant coatings [43,54]. Fire-retardant coatings prepared by adding approximately 10 wt% SiO_2_ aerogels to cement mortar can achieve a compressive strength of 3.5 MPa, a bond strength of 0.36 MPa, freeze-thaw resistance exceeding 15 cycles, and water resistance for up to 720 h, with basic conditions meeting the tunnel application environment. Such SiO_2_ aerogel mortar maintains a stable structure and good fire resistance within the critical fire conditions (<1100 °C, <1.5 h). This critical condition stems from the volume shrinkage during the crystalline phase transition of SiO_2_ aerogels, which transforms from amorphous to crystalline after 1.5 h at 1100 °C [88]. The working duration can be appropriately extended by increasing the coating thickness; for example, a 40 mm thickness can effectively withstand tunnel fires at 1100 °C for 2.5 h, with the back surface temperature at only 124 °C, protecting self-compacting concrete (SCC) from spalling and structural damage [89]. Studies have shown that the curing temperature is an important factor affecting the fire resistance of aerogel cement slurry coatings, with 50 °C being the optimal curing temperature. Within the range of 5 °C to 50 °C, increasing the curing temperature improves the fire resistance by optimizing the hydration products and microstructure; beyond 50 °C, performance decreases due to structural deterioration [90].

### 6.3. Cable and Pipeline Field

Fire-retardant coatings for cables and pipelines need to balance thermal insulation protection and cable flexibility, avoiding cracking when cables bend due to excessively hard coatings. Inorganic aerogels achieve adaptation by regulating the mechanical properties and fire-retardant efficiency of coatings.

Silica aerogels have great application potential in fire-retardant coatings for flexible cables. For example, the Harbin Institute of Technology [91] prepared a highly flexible aerogel coating with a hierarchical pore structure by compounding aerogels with aluminum dihydrogen phosphate. Its coating (3 mm thick) can maintain the back surface temperature at 220 °C when continuously heated at 1100 °C on the fire-exposed surface for 30 min, and has excellent deformability (capable of bending and twisting, etc.), with an adhesion strength to stainless steel reaching 13.6 MPa, showing great application value in fire protection fields where cables require flexibility. The polyurethane matrix has good flexibility, but the thermal insulation and fire resistance are poor. The surface-modified SiO_2_ aerogel can be combined with polyurethane to form a composite material with both flexibility and thermal insulation [56,92]. The Jiangsu Anjia Company (Suqian, China) [93] proposed an anti-cracking aerogel coating and its preparation method, which uses polyurethane as the matrix and is compounded and modified with SiO_2_ aerogels to form a core–shell structure with polyurethane coating aerogels. Combined with the cage-like cavities inherent in polyurethane, the coating film of the paint has high toughness, with a tensile strength of approximately 0.45 MPa, demonstrating its application potential as a cable fire-retardant coating. The application of aerogels in cables can also be realized through structural design; for example, the Guangxi Diecai Cable Group Co., Ltd. (Nanning, China) [94] designed a highly flexible and flame-retardant special mineral insulated fire-resistant cable and its preparation method, where SiO_2_ aerogels play an important role in lightweight reduction, fire protection, and thermal insulation. The flexibility of the cable also requires synergistic effects from components and structures such as internal inorganic fiber networks and flame-retardant and flexible organic–inorganic composite wrapping layers. This multi-layer structured cable combines fire resistance, thermal insulation, flexibility, lightweight property, and corrosion resistance, suitable for cable environments in high-risk working conditions.

### 6.4. High-Temperature Special Equipment Field

Fire-retardant coatings for high-temperature equipment in aerospace, metallurgy, and other fields need to withstand temperatures above 1000 °C. Inorganic aerogels, especially metal oxides with high-temperature resistance, have become core modified materials. Metal oxide aerogels after multi-component compounding can even withstand high temperatures of up to 1500 °C for a long time [76]. However, their inherent brittleness and the bottleneck that it is difficult for auxiliary materials to withstand high temperatures limit their development and application.

In this field, metal oxide aerogels usually need to be compounded with inorganic fibers to solve their brittleness problem. Using mullite fibers as the skeleton and ZrO_2_–SiO_2_ aerogels as “fillers”, aerogel composites with a bird’s nest–mimetic structure are prepared via vacuum impregnation. Their compressive strength can reach up to 1.05 MPa, twice that of mullite fiber blanks and ten times that of pure aerogels [68]. Some studies [73] also report that ZrO_2_–SiO_2_ aerogels, after compounding with ZrO_2_ fibers and the surface deposition of SiO_2_ films, have their compressive strength increased from the initial 0.36 MPa to 0.82 MPa. Using zirconia fiber felt as the skeleton to provide high strength and ZrO_2_–SiO_2_ aerogels as fillers, fiber felt/aerogel composites are prepared via vacuum impregnation. Their compressive strength (0.17 MPa) is six times higher than that of zirconia fiber felt, indicating that there is still much room for improving the brittleness of composites [95].

### 6.5. Wood and Lightweight Building Materials Field

Fire-retardant coatings for lightweight building materials such as wood and plastics need to balance transparency, decorativeness, and flame retardancy. Inorganic aerogels achieve performance balance through micro–nanoscale dispersion.

Silica aerogels perform excellently in this field. In intumescent fire-retardant coatings for wood fire protection, 0.45 wt% precipitated silica aerogels synergize with aluminum powder, which can reduce the peak heat release rate and increase the flame retardancy index to 2.52. The amorphous silica–aluminum gel formed through in situ cross-linking enhances the continuity of the char layer [96]. Adding 5% SiO_2_ aerogels to non-intumescent acrylic coatings for building roof fire protection can reduce the heat loss by 25% and lower the surface temperature by 7–8 °C, with better compatibility with acrylamide-containing resins, improving the coating stability [60]. Fire-retardant coatings prepared by introducing modified silica aerogels with internal hydrophobicity and external hydrophilicity into water-based resins can achieve fire resistance, moisture resistance, and thermal insulation, suitable for lightweight buildings such as wood [97]. Compounding aerogels with silicates such as sodium silicate can form a transparent intumescent fire-retardant coating, meeting the light transmission requirements of lightweight building materials on the substrate [98]; compounding aerogels with methyl azo dyes can endow fire-retardant coatings with various designed colors, meeting the decorative needs of lightweight building materials [99].

### 6.6. Application Characteristics Comparison and Selection Strategy of Inorganic Aerogels

Different inorganic aerogels exhibit differences in performance in various application fields, requiring selection strategies based on scenario requirements (Table 4). Silica aerogels are suitable for scenarios with high requirements for thermal insulation and lightweight property (e.g., steel structures and cables), but attention should be paid to hydrophobic modification in humid environments; metal oxide aerogels (Al_2_O_3_ and ZrO_2_) are preferred for high-temperature environments (e.g., special equipment and tunnels), requiring balancing density by compounding lightweight aggregates; composite aerogels have more advantages in fields requiring a high performance balance (e.g., underground engineering and lightweight building materials), and their synergistic effects can make up for the shortcomings of single aerogels.

## 7. Conclusions and Prospect

Research on aerogel-based fire-resistant coatings is transitioning from fundamental understandings to a critical phase of systematic design and engineering applications. This review has systematically delineated its developmental trajectory: commencing with an analysis of multi-faceted flame-retardant and thermal insulation mechanisms, it established the theoretical foundation for material functionalization. Subsequently, by systematically comparing the intrinsic properties of different aerogel types, it clarified the comprehensive advantages and performance limitations of silica-based aerogels under the current technological conditions, thereby providing a scientific basis for rational material selection. Confronted with a series of technical bottlenecks exposed during the coating process—including nanopore preservation, interfacial bonding, mechanical enhancement, application compatibility, and scalable cost—the research community has developed corresponding composite modification strategies. These encompass surface engineering, structural design, workability regulation, and low-cost fabrication, which have demonstrated significant potential for synergistic enhancement.

Looking forward, the continued advancement of aerogel fire-resistant coatings relies on breakthroughs across multiple dimensions: On the materials front, it is necessary to move beyond the reliance on single SiO_2_ systems and explore differentiated application scenarios for novel aerogels such as carbon-based and biomass-derived types. Technologically, focused efforts are required to overcome core industrialization challenges including long-term interfacial stability, environmentally friendly formulations, and absolute cost control. Functionally, developing next-generation coatings incorporating advanced intelligent features—such as damage self-sensing and self-healing capabilities—will be a pivotal direction for leading the field’s future development. In summary, by advancing the innovation chain spanning “mechanism–material–design–application”, aerogel-based fire-resistant coatings not only provide a transformative solution for enhancing engineering fire safety, but are also poised to play an increasingly significant role in green building and sustainable development.

## Figures and Tables

**Table 1:** Comprehensive comparison of characteristics of different types of aerogels.

Category	Characteristic	Silica-Based Aerogels	Metal Oxide Aerogels	Carbon-Based Aerogels	Bio-Based Aerogels
Physical properties	Density (mg cm^−3^)	88.5–120 [16,24,25]	102–181 [27,28,29,30]	4.6–18.7 [33,34,35,36]	11.8–52 [18,39,40]
Thermal performance	Thermal conductivity (mW·m^−1^·K^−1^)	18–21 [16,24]	~28 [29]	12.7–16.5 [33,36]	27.2–35.4 [18,39,40,41]
	Thermal stability	650~800 °C [31]	>1000 °C [31]	~570 °C [33]	Burnable
Mechanical performance	Mechanical property	Brittle compression modulus: 0.62 MPa [25]	Brittle compressive stress: 0.02–0.23 MPa [27,30]	Elasticity/toughness [33,34,35,36]	Toughness compression modulus: 18.2–25 MPa [40,42]
Surface and compatibility	Surface	Hydrophilic	Hydrophilic	Hydrophobic/inert	Strong hydrophilic
	Compatibility in the coating	High (powder/slurry)	Medium–low (powdering is a challenge)	Low (mostly blocks)	Medium (powder)
Application characteristics	Intrinsic flame retardancy	High (non-combustible)	High (non-combustible)	Low (burnable in air)	Low (burnable in air)
	Cost	Medium to high (continued decline)	Extreme high	High	High
	Technical maturity	High (commercially available)	Low (special field)	Low (other fields)	Low (basic research stage)

**Table 2:** Effects of surface modification strategies of aerogels on properties of composites.

Strategy	Material System (Aerogel@resin)	Pore Structure and Interface Bonding	Mechanical Properties (Modified vs. Unmodified)	Dispersion Stability	Thermal Conductivity mW·m^−1^·K^−1^ (Modified vs. Unmodified)	Ref.
Hydrophobic-modified	SiO_2_@epoxy resin	/	/	Good dispersion—SEM	/	[49]
	SiO_2_@acrylic emulsion	Indistinct dipping (density decreases with the aerogel content)	Tensile strength: 0.183 MPa	Good dispersion	71.2 vs. /	[55]
	SiO_2_@silicone resin	SEM—Good combination; dipping unknown	/	Good dispersion—SEM	100 vs. /	[50]
	SiO_2_@polyurethane	SEM—Good combination; dipping unknown	/	Good dispersion—SEM	130 vs. /	[56]
Dual-modified	SiO_2_@epoxy silicon resin	TEM—dipping	/	Good dispersion—SEM	/	[51]
	SiO_2_@acrylic emulsion	SEM—Good combination; indistinct dipping	/	Good dispersion—SEM	90 vs. 150	[52]
	SiO_2_-acrylic emulsion	/	Compressive strength: 3.44 vs. 0.66 MPa	/	/	[53]
	SiO_2_@epoxy resin	SEM—Good combination; dipping unknown	Tensile strength: 51.96 vs. 36.41 MPa	Good dispersion—SEM	/	[57]
KH550	SiO_2_@epoxy resin	SEM—Good combination; dipping unknown	Compressive strength: 63.2 MPa	Good dispersion—SEM	/	[58]
	SiO_2_@cement mortar	SEM—Good combination; indistinct dipping	Compressive strength: 2.15 MPa	Good dispersion—SEM	152 vs. /	[43]
KH570	SiO_2_-cement mortar	SEM—Good combination; indistinct dipping	Compressive strength: 17.13 vs. 2.83 MPa	Good dispersion	1061 vs. 1120	[54]

**Table 4:** Performance comparison of and selection suggestions for inorganic aerogels in various application fields.

Application Fields	Resin/Coating Matrix	Preferable Aerogel Type	Key Index
Construction steel structure	Epoxy resin [21,84,85] Silicon resin [49] Gypsum [86]	SiO_2_ aerogels; composite aerogels	Resin compatibility, adhesive strength, etc.
Tunnel and underground engineering	Cement [43,53]	SiO_2_ aerogels; composite aerogels	Environmental friendliness, water resistance, etc.
Cable and pipeline	Polyurethane [90,91,92,93]	SiO_2_ aerogels; composite aerogels	Flexibility, etc.
High-temperature equipment	/	ZrO_2_–SiO_2_ aerogels; Al_2_O_3_–SiO_2_ aerogels	Structural stability at high temperature, adhesive strength, etc.
Wood and lightweight building	Acrylic resin [59] Polyurethane [96] Sodium silicate [97]	SiO_2_ aerogels; composite aerogels	Transparency, color, etc.

## Data Availability

No new data were created or analyzed in this study. Data sharing is not applicable to this article.
